# l-Leucylglycylglycine

**DOI:** 10.1107/S1600536813008490

**Published:** 2013-04-05

**Authors:** Masanori Ootaki, Yukino Nawa, Tomoko Hiroi, Hiroaki Matsui, Yoko Sugawara

**Affiliations:** a Institute of Radioisotope Research, St. Marianna University Graduate School of Medicine, 2-16-1 Sugao, Miyamae-ku, Kawasaki, Kanagawa 216-8511, Japan; b Department of Molecular and Behavioral Neuroscience, St. Marianna University Graduate School of Medicine, 2-16-1 Sugao, Miyamae-ku, Kawasaki, Kanagawa 216-8511, Japan; c School of Science, Kitasato University, 1-15-1 Kitasato, Minami-ku, Sagamihara, Kanagawa 252-0373, Japan

## Abstract

In the title compound, C_10_H_19_N_3_O_4_, the N- and C-termini are protonated and ionized, respectively, and the mol­ecule forms a zwitterion. The main chain is in a folded form. In the crystal, the N-terminal –NH_3_^+^ group hydrogen bonds to three C-terminal –COO groups and one carbonyl O atom, forming a three-dimensional network. In addition, an N—H⋯O hydrogen bond between the amide groups of the middle glycine residue and a C—H⋯O inter­action continue along the *a-*axis direction. The side chains of the leucyl residues form a hydro­phobic region along the *a* axis.

## Related literature

For related structures of l-leucylglycylglycine, see: Goswami *et al.* (1977 ▶); Srikrishnan & Parthasarathy (1987 ▶); Kiyotani & Sugawara (2012 ▶).
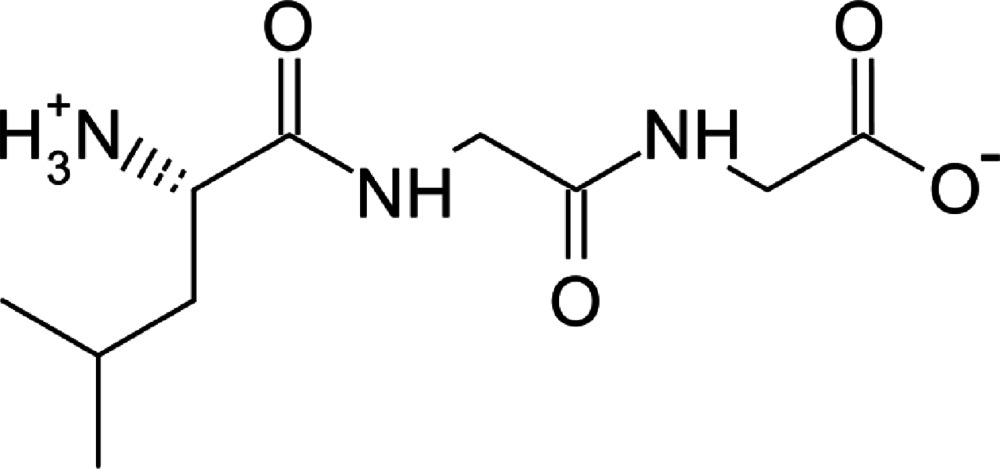


## Experimental

### 

#### Crystal data

C_10_H_19_N_3_O_4_
*M* *_r_* = 245.28
Orthorhombic, 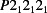

*a* = 5.391 (5) Å
*b* = 11.742 (10) Å
*c* = 19.975 (16) Å
*V* = 1264.4 (19) Å^3^
*Z* = 4
Mo *K*α radiation
μ = 0.10 mm^−1^
*T* = 173 K
0.48 × 0.18 × 0.08 mm

#### Data collection

Rigaku Mercury CCD area-detecter diffractometer
9374 measured reflections
2887 independent reflections
2200 reflections with *I* > 2σ(*I*)
*R*_int_ = 0.070

#### Refinement

*R*[*F* ^2^ > 2σ(*F* ^2^)] = 0.038
*wR*(*F* ^2^) = 0.087
*S* = 0.96
2887 reflections
176 parameters
3 restraints
H atoms treated by a mixture of independent and constrained refinement
Δρ_max_ = 0.14 e Å^−3^
Δρ_min_ = −0.17 e Å^−3^

### 

Data collection: *CrystalClear* (Rigaku, 2006 ▶); cell refinement: *CrystalClear*; data reduction: *CrystalClear*; program(s) used to solve structure: *SHELXS97* (Sheldrick, 2008 ▶); program(s) used to refine structure: *SHELXL97* (Sheldrick, 2008 ▶); molecular graphics: *Mercury* (Macrae *et al.*, 2006 ▶); software used to prepare material for publication: *SHELXL97* (Sheldrick, 2008 ▶) and *Yadokari-XG 2009* (Kabuto *et al.*, 2009 ▶).

## Supplementary Material

Crystal structure: contains datablock(s) I, global. DOI: 10.1107/S1600536813008490/is5257sup1.cif

Structure factors: contains datablock(s) I. DOI: 10.1107/S1600536813008490/is5257Isup2.hkl

Supplementary material file. DOI: 10.1107/S1600536813008490/is5257Isup3.cml

Additional supplementary materials:  crystallographic information; 3D view; checkCIF report

## Figures and Tables

**Table 1:** Hydrogen-bond geometry (Å, °)

*D*—H⋯*A*	*D*—H	H⋯*A*	*D*⋯*A*	*D*—H⋯*A*
N1—H1⋯O3^i^	0.97 (3)	1.78 (3)	2.743 (2)	168 (2)
N1—H2⋯O4^ii^	0.92 (2)	1.94 (2)	2.822 (2)	160 (2)
N1—H2⋯O3^ii^	0.92 (2)	2.52 (2)	3.260 (2)	138 (2)
N1—H3⋯O1^iii^	0.89 (2)	2.45 (2)	3.031 (2)	123 (2)
N1—H3⋯O3^iv^	0.89 (2)	2.05 (2)	2.870 (2)	151 (2)
N2—H5⋯O2^v^	0.83 (2)	2.05 (2)	2.832 (2)	155 (2)
C1—H4⋯O1^v^	1.00	2.33	3.269 (2)	155

Symmetry codes: (i) 
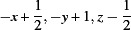
; (ii) 
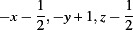
; (iii) 
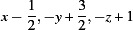
; (iv) 
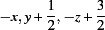
; (v) 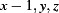.

**Fig. 1.**
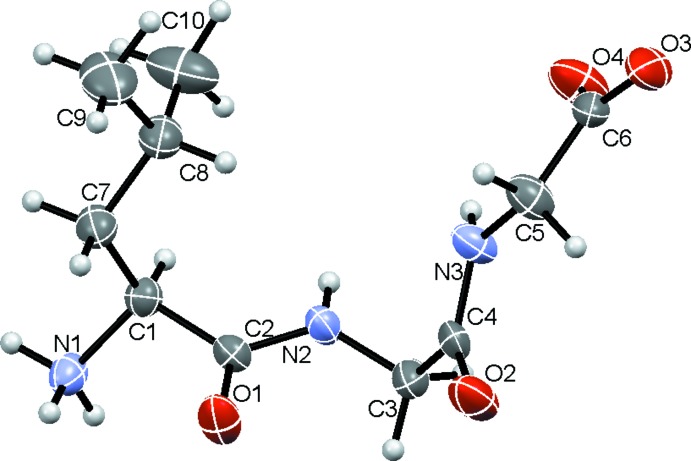
View of the title compound with the atomic numbering scheme. Displacement ellipsoids are drawn at 50% probability level and H atoms are shown as small spheres of arbitrary radii.

**Fig. 2.**
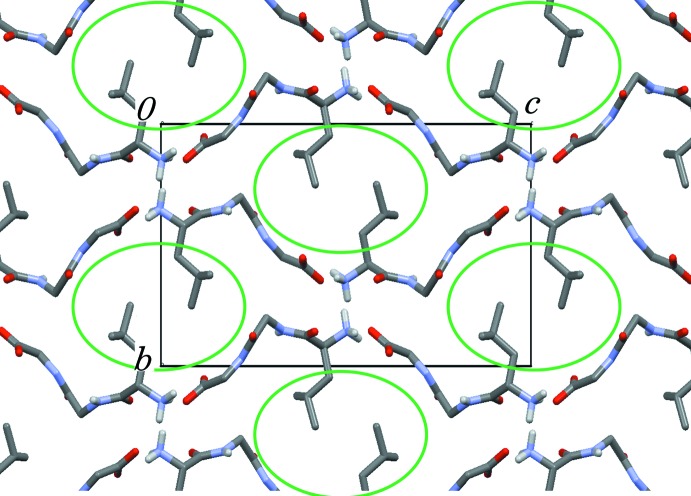
Packing diagram of the title compound viewed along the a axis. Hydrophobic columns are indicated by green circles.

**Fig. 3.**
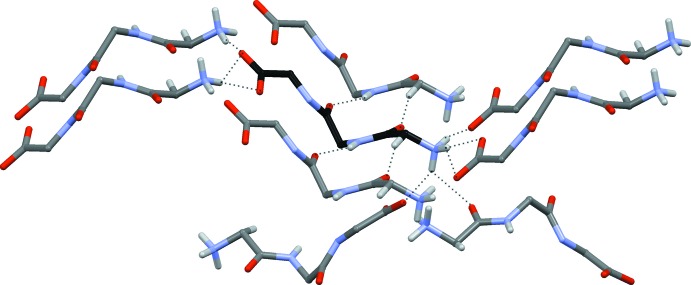
Hydrogen bonding scheme around the molecule, whose carbon atoms are colored with black. Hydrogen bonds are indicated by dotted lines. Side-chain atoms of the leucyl residues have been omitted for clarity.

## supplementary crystallographic information

### Comment

A moderately large number of oligopeptides are biologically active, and their structures are investigated to facilitate the determination of the possible conformations of oligopeptide and polypeptide chains. The N-terminus and C-terminus of *L*-leucylglycylglycine (*L*-LGG) are protonated and ionized, respectively, and the molecule is a zwitterion (Fig. 1). The main chain is in a folded form. The torsion angles of C2—N2—C3—C4 and N2—C3—C4—N3 are -87.54 (18)° and -52.78 (19)°, respectively. The N-terminal –NH_3_^+^ groups and the C-terminal –COO^-^ groups form hydrogen bond networks (Fig. 2 & Fig. 3). One of the hydrogen atoms of the –NH_3_ group forms a three-centered hydrogen bond with the carboxyl and carboxyl oxygen atoms. In addition, intermolecular hydrogen bonds among amide groups (NH···O═C) are formed along the *a* axis. The hydrophobic region composed of leucyl side chains is surrounded by hydrophilic parts, and forms a column along the *a* axis.

In the case of the *L*-leucylglycylglycylglycine (Srikrishnan & Parthasarathy, 1987), the main chain is a folded form, and hydrophobic columns are formed along the *a* axis as in the case of *L*-LGG. On the other hand, the main chain of *D*,*L*-leucylglycylglycine (*D*,*L*-LGG) (Goswami *et al.*, 1977) is in a nearly all-*trans* form expect the N-terminus. The main chains align parallel to the *b* axis in a head-to-tail manner and a β-sheetlike structure is formed parallel to the *bc* plane. The hydrophobic regions of the leucyl side chains and the hydrophilic regions are aligned alternately along the *a* axis. As in the case of *D*,*L*-LGG, the main chain in *L*-leucylglycine 0.67 hydrate (Kiyotani & Sugawara, 2012) is in a extended form, and hydrophobic and hydrophilic regions are aligned alternately along the *c* axis.

### Experimental

*L*-Leucylglycylglycine was purchased from Bachem Inc. Single crystals were obtained from an aqueous solution.

### Refinement

H atoms were placed in calculated positions with C—H = 0.98 Å (CH_3_), 0.99 Å (CH_2_) or 1.00 Å (CH) and refined in a riding mode with *U*_iso_(H) = 1.2*U*_eq_(C). The other H atoms were placed in a difference Fourier map. The N-terminal H atoms were restrained to N—H = 0.87 (4) Å during refinements. The absolute configuration was known for the purchased material.

### Crystal data

**Table:**

C_10_H_19_N_3_O_4_	*F*(000) = 528
*M**_r_* = 245.28	*D*_x_ = 1.288 Mg m^−^^3^
Orthorhombic, *P*2_1_2_1_2_1_	Mo *K*α radiation, λ = 0.71070 Å
Hall symbol: P 2ac 2ab	Cell parameters from 949 reflections
*a* = 5.391 (5) Å	θ = 7.6–17.5°
*b* = 11.742 (10) Å	µ = 0.10 mm^−^^1^
*c* = 19.975 (16) Å	*T* = 173 K
*V* = 1264.4 (19) Å^3^	Block Rod, colorless
*Z* = 4	0.48 × 0.18 × 0.08 mm

### Data collection

**Table:**

Rigaku Mercury CCD area-detecter diffractometer	2200 reflections with *I* > 2σ(*I*)
Radiation source: rotating anode	*R*_int_ = 0.070
Graphite monochromator	θ_max_ = 27.5°, θ_min_ = 3.5°
ω scans	*h* = −6→6
9374 measured reflections	*k* = −15→15
2887 independent reflections	*l* = −21→25

### Refinement

**Table:**

Refinement on *F*^2^	Primary atom site location: structure-invariant direct methods
Least-squares matrix: full	Secondary atom site location: difference Fourier map
*R*[*F*^2^ > 2σ(*F*^2^)] = 0.038	Hydrogen site location: inferred from neighbouring sites
*wR*(*F*^2^) = 0.087	H atoms treated by a mixture of independent and constrained refinement
*S* = 0.96	*w* = 1/[σ^2^(*F*_o_^2^) + (0.0374*P*)^2^] where *P* = (*F*_o_^2^ + 2*F*_c_^2^)/3
2887 reflections	(Δ/σ)_max_ = 0.012
176 parameters	Δρ_max_ = 0.14 e Å^−^^3^
3 restraints	Δρ_min_ = −0.17 e Å^−^^3^

### Special details

**Table:**

Geometry. All e.s.d.'s (except the e.s.d. in the dihedral angle between two l.s. planes) are estimated using the full covariance matrix. The cell e.s.d.'s are taken into account individually in the estimation of e.s.d.'s in distances, angles and torsion angles; correlations between e.s.d.'s in cell parameters are only used when they are defined by crystal symmetry. An approximate (isotropic) treatment of cell e.s.d.'s is used for estimating e.s.d.'s involving l.s. planes.
Refinement. Refinement of *F*^2^ against ALL reflections. The weighted *R*-factor *wR* and goodness of fit *S* are based on *F*^2^, conventional *R*-factors *R* are based on *F*, with *F* set to zero for negative *F*^2^. The threshold expression of *F*^2^ > σ(*F*^2^) is used only for calculating *R*-factors(gt) *etc*. and is not relevant to the choice of reflections for refinement. *R*-factors based on *F*^2^ are statistically about twice as large as those based on *F*, and *R*- factors based on ALL data will be even larger.

### Fractional atomic coordinates and isotropic or equivalent isotropic displacement parameters (Å^2^)

**Table:**

	*x*	*y*	*z*	*U*_iso_*/*U*_eq_	
N1	−0.1744 (3)	0.64554 (12)	0.49670 (7)	0.0348 (3)	
N2	−0.0484 (3)	0.64749 (12)	0.67001 (6)	0.0315 (3)	
H5	−0.195 (4)	0.6402 (15)	0.6825 (9)	0.036 (5)*	
N3	0.1814 (3)	0.52895 (12)	0.78349 (7)	0.0362 (3)	
H8	0.022 (4)	0.5354 (16)	0.7880 (9)	0.048 (6)*	
O1	0.2239 (2)	0.64148 (11)	0.58454 (5)	0.0415 (3)	
O2	0.5246 (2)	0.62376 (11)	0.75146 (6)	0.0485 (3)	
C1	−0.1911 (3)	0.58011 (12)	0.56075 (8)	0.0290 (3)	
H4	−0.3570	0.5921	0.5820	0.035*	
C2	0.0137 (3)	0.62595 (12)	0.60611 (7)	0.0273 (3)	
C3	0.1320 (3)	0.69772 (14)	0.71523 (8)	0.0368 (4)	
H6	0.0422	0.7431	0.7493	0.044*	
H7	0.2381	0.7507	0.6894	0.044*	
C4	0.2975 (3)	0.61277 (13)	0.75092 (7)	0.0314 (4)	
C5	0.3059 (3)	0.45098 (16)	0.82883 (9)	0.0406 (4)	
H9	0.4550	0.4883	0.8477	0.049*	
H10	0.3604	0.3826	0.8038	0.049*	
C6	0.1333 (4)	0.41541 (13)	0.88559 (8)	0.0354 (4)	
O3	0.2277 (3)	0.35522 (11)	0.93090 (6)	0.0536 (4)	
O4	−0.0855 (3)	0.44525 (12)	0.88306 (7)	0.0545 (4)	
C7	−0.1526 (3)	0.45349 (13)	0.54488 (8)	0.0374 (4)	
H11	−0.2711	0.4312	0.5094	0.045*	
H12	0.0168	0.4434	0.5267	0.045*	
C8	−0.1855 (4)	0.37232 (14)	0.60441 (9)	0.0413 (4)	
H13	−0.0679	0.3959	0.6406	0.050*	
C9	−0.1199 (5)	0.25116 (15)	0.58278 (10)	0.0636 (7)	
H14	−0.1379	0.1995	0.6210	0.076*	
H15	0.0518	0.2492	0.5667	0.076*	
H16	−0.2316	0.2272	0.5467	0.095*	
C10	−0.4475 (5)	0.3769 (2)	0.63228 (13)	0.0715 (7)	
H17	−0.5664	0.3609	0.5964	0.086*	
H18	−0.4796	0.4529	0.6507	0.086*	
H19	−0.4656	0.3199	0.6678	0.086*	
H1	−0.011 (5)	0.636 (2)	0.4768 (12)	0.089 (9)*	
H2	−0.284 (5)	0.6147 (19)	0.4666 (12)	0.076 (7)*	
H3	−0.202 (4)	0.7193 (16)	0.5047 (11)	0.058 (6)*	

### Atomic displacement parameters (Å^2^)

**Table:**

	*U*^11^	*U*^22^	*U*^33^	*U*^12^	*U*^13^	*U*^23^
N1	0.0420 (9)	0.0301 (7)	0.0322 (7)	−0.0012 (7)	−0.0133 (7)	0.0028 (6)
N2	0.0312 (8)	0.0404 (8)	0.0228 (6)	−0.0052 (7)	0.0020 (6)	0.0025 (6)
N3	0.0288 (8)	0.0458 (8)	0.0341 (7)	−0.0020 (7)	0.0000 (6)	0.0152 (6)
O1	0.0248 (6)	0.0666 (8)	0.0332 (6)	−0.0019 (6)	0.0030 (5)	0.0004 (6)
O2	0.0387 (8)	0.0668 (9)	0.0399 (7)	−0.0099 (7)	0.0061 (5)	0.0128 (6)
C1	0.0284 (8)	0.0304 (7)	0.0282 (8)	0.0006 (7)	−0.0021 (7)	0.0001 (6)
C2	0.0272 (8)	0.0294 (8)	0.0253 (7)	0.0018 (7)	0.0018 (6)	0.0038 (6)
C3	0.0496 (12)	0.0340 (8)	0.0268 (8)	−0.0083 (8)	−0.0054 (8)	0.0034 (7)
C4	0.0375 (10)	0.0396 (9)	0.0172 (7)	−0.0051 (8)	0.0031 (7)	0.0006 (6)
C5	0.0365 (10)	0.0480 (10)	0.0373 (9)	0.0046 (9)	0.0010 (8)	0.0148 (8)
C6	0.0498 (11)	0.0301 (8)	0.0264 (8)	−0.0120 (8)	−0.0017 (8)	0.0028 (7)
O3	0.0698 (9)	0.0532 (7)	0.0379 (7)	−0.0196 (7)	−0.0195 (7)	0.0220 (6)
O4	0.0427 (8)	0.0644 (9)	0.0562 (8)	0.0010 (7)	0.0156 (7)	0.0178 (7)
C7	0.0482 (11)	0.0303 (8)	0.0338 (9)	0.0007 (8)	−0.0012 (8)	−0.0004 (7)
C8	0.0532 (11)	0.0325 (8)	0.0381 (9)	−0.0025 (9)	−0.0036 (8)	0.0037 (8)
C9	0.096 (2)	0.0342 (10)	0.0602 (14)	0.0078 (10)	0.0029 (13)	0.0088 (9)
C10	0.0713 (16)	0.0552 (13)	0.0882 (17)	−0.0017 (12)	0.0223 (13)	0.0234 (12)

### Geometric parameters (Å, º)

**Table:**

N1—C1	1.495 (2)	C5—C6	1.525 (3)
N1—H1	0.97 (2)	C5—H9	0.9900
N1—H2	0.92 (2)	C5—H10	0.9900
N1—H3	0.894 (19)	C6—O4	1.232 (3)
N2—C2	1.344 (2)	C6—O3	1.256 (2)
N2—C3	1.452 (2)	C7—C8	1.534 (2)
N2—H5	0.83 (2)	C7—H11	0.9900
N3—C4	1.336 (2)	C7—H12	0.9900
N3—C5	1.452 (2)	C8—C10	1.519 (3)
N3—H8	0.87 (2)	C8—C9	1.528 (3)
O1—C2	1.226 (2)	C8—H13	1.0000
O2—C4	1.231 (2)	C9—H14	0.9800
C1—C2	1.526 (2)	C9—H15	0.9800
C1—C7	1.534 (2)	C9—H16	0.9800
C1—H4	1.0000	C10—H17	0.9800
C3—C4	1.516 (2)	C10—H18	0.9800
C3—H6	0.9900	C10—H19	0.9800
C3—H7	0.9900		
			
C1—N1—H1	110.2 (15)	C6—C5—H9	109.5
C1—N1—H2	108.6 (14)	N3—C5—H10	109.5
H1—N1—H2	105 (2)	C6—C5—H10	109.5
C1—N1—H3	109.6 (14)	H9—C5—H10	108.1
H1—N1—H3	110 (2)	O4—C6—O3	125.31 (17)
H2—N1—H3	113 (2)	O4—C6—C5	118.43 (16)
C2—N2—C3	120.03 (15)	O3—C6—C5	116.25 (18)
C2—N2—H5	120.0 (13)	C8—C7—C1	115.23 (14)
C3—N2—H5	119.4 (13)	C8—C7—H11	108.5
C4—N3—C5	123.47 (17)	C1—C7—H11	108.5
C4—N3—H8	116.8 (13)	C8—C7—H12	108.5
C5—N3—H8	116.7 (13)	C1—C7—H12	108.5
N1—C1—C2	106.45 (13)	H11—C7—H12	107.5
N1—C1—C7	108.24 (13)	C10—C8—C9	110.59 (17)
C2—C1—C7	111.49 (13)	C10—C8—C7	111.68 (16)
N1—C1—H4	110.2	C9—C8—C7	109.41 (15)
C2—C1—H4	110.2	C10—C8—H13	108.4
C7—C1—H4	110.2	C9—C8—H13	108.4
O1—C2—N2	122.41 (14)	C7—C8—H13	108.4
O1—C2—C1	120.84 (14)	C8—C9—H14	109.5
N2—C2—C1	116.75 (14)	C8—C9—H15	109.5
N2—C3—C4	114.79 (14)	H14—C9—H15	109.5
N2—C3—H6	108.6	C8—C9—H16	109.5
C4—C3—H6	108.6	H14—C9—H16	109.5
N2—C3—H7	108.6	H15—C9—H16	109.5
C4—C3—H7	108.6	C8—C10—H17	109.5
H6—C3—H7	107.5	C8—C10—H18	109.5
O2—C4—N3	122.62 (16)	H17—C10—H18	109.5
O2—C4—C3	121.36 (15)	C8—C10—H19	109.5
N3—C4—C3	115.97 (17)	H17—C10—H19	109.5
N3—C5—C6	110.74 (16)	H18—C10—H19	109.5
N3—C5—H9	109.5		
			
C3—N2—C2—O1	4.0 (2)	N2—C3—C4—O2	129.75 (17)
C3—N2—C2—C1	−176.27 (13)	N2—C3—C4—N3	−52.78 (19)
N1—C1—C2—O1	−45.43 (19)	C4—N3—C5—C6	147.06 (16)
C7—C1—C2—O1	72.41 (19)	N3—C5—C6—O4	7.0 (2)
N1—C1—C2—N2	134.83 (14)	N3—C5—C6—O3	−174.41 (14)
C7—C1—C2—N2	−107.33 (16)	N1—C1—C7—C8	−175.12 (14)
C2—N2—C3—C4	−87.54 (18)	C2—C1—C7—C8	68.14 (19)
C5—N3—C4—O2	7.5 (2)	C1—C7—C8—C10	62.1 (2)
C5—N3—C4—C3	−169.93 (15)	C1—C7—C8—C9	−175.13 (17)

### Hydrogen-bond geometry (Å, º)

**Table:**

*D*—H···*A*	*D*—H	H···*A*	*D*···*A*	*D*—H···*A*
N1—H1···O3^i^	0.97 (3)	1.78 (3)	2.743 (2)	168 (2)
N1—H2···O4^ii^	0.92 (2)	1.94 (2)	2.822 (2)	160 (2)
N1—H2···O3^ii^	0.92 (2)	2.52 (2)	3.260 (2)	138 (2)
N1—H3···O1^iii^	0.89 (2)	2.45 (2)	3.031 (2)	123 (2)
N1—H3···O3^iv^	0.89 (2)	2.05 (2)	2.870 (2)	151 (2)
N2—H5···O2^v^	0.83 (2)	2.05 (2)	2.832 (2)	155 (2)
C1—H4···O1^v^	1.00	2.33	3.269 (2)	155

Symmetry codes: (i) −*x*+1/2, −*y*+1, *z*−1/2; (ii) −*x*−1/2, −*y*+1, *z*−1/2; (iii) *x*−1/2, −*y*+3/2, −*z*+1; (iv) −*x*, *y*+1/2, −*z*+3/2; (v) *x*−1, *y*, *z*.
